# Erianin induces triple-negative breast cancer cells apoptosis by activating PI3K/Akt pathway

**DOI:** 10.1042/BSR20210093

**Published:** 2021-06-10

**Authors:** Yonggang Xu, Rong Fang, Jie Shao, Zihao Cai

**Affiliations:** 1 Department of General Surgery, Huashan Hospital, Fudan University, Shanghai 200040, P.R. China; 2 School of Medicine, Ningbo University, Ningbo 315020, P.R. China

**Keywords:** breast cancer;, cell apoptosis, cell proliferation, erianin, PI3K/Akt pathway

## Abstract

**Background:** Triple-negative breast cancer (TNBC) is a refractory subtype of breast cancer, 25–30% of which have dysregulation in the PI3K/AKT pathway. The present study investigated the anticancer effect of erianin on TNBC cell line and its underlying mechanism.

**Methods:** After treatment with erianin, MTT assay was employed to determine the MDA-MB-231 and EFM-192A cell proliferation, the nucleus morphological changes were observed by DAPI staining. The cell cycle and apoptotic proportion were detected by flow cytometry. Western blot was performed to determine the cell cycle and apoptosis-related protein expression and PI3K pathways. Finally, the antiproliferative activity of erianin was further confirmed by adding or not adding PI3K agonists SC79.

**Results:** Erianin inhibited the proliferation of MDA-MB-231 and EFM-192A cells in a dose-dependent manner, the IC_50_ were 70.96 and 78.58 nM, respectively. Erianin could cause cell cycle arrest at the G_2_/M phase, and the expressions of p21 and p27 were up-regulated, while the expressions of CDK1 and Cyclin B1 were down-regulated. Erianin also induced apoptosis via the mitochondrial pathway, with the up-regulation of the expression of Cyto C, PARP, Bax, active form of Caspase-3, and Caspase-9. Furthermore, p-PI3K and p-Akt expression were down-regulated by erianin. After co-incubation with SC79, the cell inhibition rate of erianin was decreased, which further confirmed that the attenuated PI3K/Akt pathway was relevant to the pro-apoptotic effect of erianin.

**Conclusions:** Erianin can inhibit the proliferation of TNBC cells and induce cell cycle arrest and apoptosis, which may ascribe to the abolish the activation of the PI3K/Akt pathway.

## Background

Breast cancer is one of the most common epidermal cell tumors, which seriously threaten women’s health. The combination of surgery and other adjuvant therapies, which contains chemotherapy, radiation therapy, and endocrine therapy, are major treatments to cure breast cancer. Although the above comprehensive therapy could achieve a certain therapeutic effect, it would have limited effects on Triple-Negative Breast Cancer (TNBC). An important way to find lead compounds is to acquire the biologically active products in the natural plants. The research on the exploring and application of activity substrate extraction from natural plants, especially the TNBC, is getting increasing attention. From 1981 to 2002, statistical data showed that 74% of antitumor drugs approved by the FDA were derived from natural products or their derivatives [1], such as paclitaxel, camptothecin, podophyllotoxin etc. *Dendrobium*, a perennial herb of orchidaceae, is a traditional Chinese medicine. A large number of pharmacology studies on the *Dendrobium* species have been explored, which have shown that *Dendrobium* has multiplex effects on anti-aging [2], enhancing immunity, hepatoprotective [3], antifibrotic [4], antiviral [5], antifungal [6], antioxidant [7], antiplatelet aggregation [8], antidiabetic [9], especially antitumor [10–13], which has caught more and more attention recently.

Erianin, a bibenzyl compound named 2-methoxy-5-[2-(3,4,5-trimethoxy phenyl) ethyl]-phenol, is one of the active compounds isolated from *Dendrobium* with multiple antitumor activity, has shown its antitumor activity in lung cancer [14], liver cancer [15], oral cancer [16], nasopharyngeal carcinoma [17], bladder cancer [18] osteosarcoma [19], and cervical cancer [20]; moreover it also has anti-angiogenesis effects [21]. However, the molecular mechanism and the drug targets are largely unknown. Besides, little is known about the antitumor activity of erianin on human breast cancer cells [22,23]. Previous studies have shown that *Dendrobium candidum* extracts could inhibit breast cancer cells MCF-7 and induce cell cycle arrest [23]; further, it has reported that erianin could inhibit the growth of the breast cancer cells line T47D proliferation and migration [22], and can also induce apoptosis, but the specific mechanism still needs to be further elucidated. In the present study, we aim to investigate the effects of erianin on cell cycle and apoptosis of TNBC cells *in vitro*, and its underlying mechanism.

## Methods

### Reagents and cell lines

Human breast cancer cell line MDA-MB-231 was purchased from Cell Bank of Shanghai Institute of Biochemistry and Cell Biology (Shanghai, China), and EFM-192A was from Zhejiang Ruyao Biology Co., Ltd (Zhejiang, China). Cells were cultured with RPMI1640 medium (GIBCO) containing 10% FBS (GIBCO). Akt agonists SC79 was purchased from Sigma company.

### MTT assay

Cells were seeded at a density of 1 × 10^4^ per well in 96-well plates and cultured at 37°C in a 5% CO_2_ incubator for 48 h. Then, cells were cultured with medium diluted with erianin for another 24 h, the final concentrations were at 0, 10, 20, 40, 80, 160, 320 nM separately. Four wells were seeded at each concentration, and A570 was measured using MTT Cell Proliferation and Cytotoxicity Assay Kit (Sigma) according to the manufacturer’s protocol. The inhibitory concentration IC_50_ was calculated and the experiments were repeated three times.

### DAPI staining

Cells were cultured to grow to 70% density, treated with erianin at final concentration to 40, 80, 160 nM for 24 h. The cells were fixed with 4% paraformaldehyde for 30 min, stained by 1 μg/ml DAPI (Sigma) for 10 min. Cell morphology and apoptosis were detected under fluorescent microscopy.

### Flow cytometry

In total, 5 × 10^5^ MDA-MB-231 cells were seeded in a 6-cm-dish. Then erianin at 0, 40, 80, 160 nM were administrated for 24 h, the cells were collected and resuspended with 500 μl binding buffer at room temperature. FITC-labeled Annexin V antibody (BD) was added and incubated for 15 min at room temperature in the dark, followed by PI treatment for 5 min before performing flow cytometry. Then apoptotic cells were detected by Flow Cytometer (Attune NxT, Life Technology) and data were analyzed with FlowJo 7.6 software (TreeStar Inc).

### Western blot

Harvested cells were washed twice with phosphate-buffered saline (PBS), and proteins were extracted using 1× loading lysis buffer. Equal amounts of protein, as measured by the BCA protein assay, were separated by sodium dodecyl sulfate/polyacrylamide gel electrophoresis (SDS/PAGE) and transferred on to a polyvinylidenedifluoride (PVDF) membrane (Immobilon P, Millipore). The membrane was sealed in 5% skim milk for 1 h, and then incubated with primary antibody for 2 h. After washing with TBST for three times, it was incubated with horseradish peroxidase-bound secondary antibody for 2 h. Anti-PARP, anti-Caspase-3, anti-Caspase-9, anti-cyto C, anti-Bcl-2, anti-Bax, anti-PI3K, anti-p-PI3K, anti-Akt, anti-p-Akt, anti-CDK1, anti-Cyclin B1, anti-Cyclin A, anti-p21, anti-p27, anti-GAPDH, HRP-conjugated goat anti-rabbit IgG, and HRP-conjugated rabbit anti-mouse IgG antibodies were purchased from Cell Signaling Technology. After cleaning with TBST, the Universal ECL immunoblotting chemiluminescence solution (Vazyme, China) was used to display the bands, and relative expression of protein was analyzed by ImageJ.

### Statistical analysis

Statistical analysis and scientific mapping of experimental data were performed using GraphPad Prism 8 software and independent *t* tests were used for pairwise comparison. *P*<0.05 was considered to have significant difference.

## Results

### Erianin can inhibit cell proliferation and influence cell morphology in a dose-dependent way

**Figure 1:**
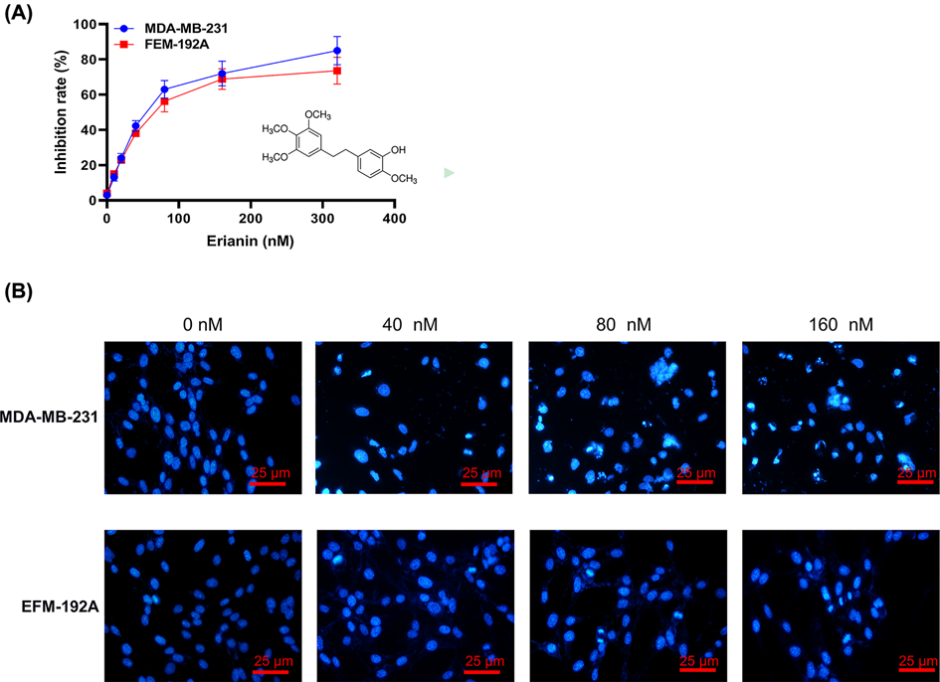
Erianin can inhibit cell proliferation and influence cell morphology in a dose-dependent way (**A**) MTT results of MDA-MB-231 and EFM-192A cells with erianin (lower right shows the structure of erianin) treatment at different concentrations. (**B**) DAPI staining of MDA-MB-231 and EFM-192A cells treated with erianin for 24 h.

To address the function of erianin on MDA-MB-231 and EFM-192A cells growth, MTT assay and DAPI staining were performed. MDA-MB-231 and EFM-192A cells’ proliferation was inhibited with erianin treatment in a dose-dependent way as shown in MTT analysis, with IC_50_ at 70.96 and 78.58 nM effective inhibition concentration at nanomolar level (Figure 1A). In addition, typical apoptotic morphology (chromatin condensation and fracture) was detected in MDA-MB-231 cells nucleus treated with 40, 80, or 160 nM erianin for 24 h in DAPI staining, indicating erianin may induce MDA-MB-231 cells apoptosis in a dose-dependent way (Figure 1B).

### Erianin induces cell cycle arrest at G_2_/M phase and influences cell cycle-related protein expression in a dose-dependent way

**Figure 2:**
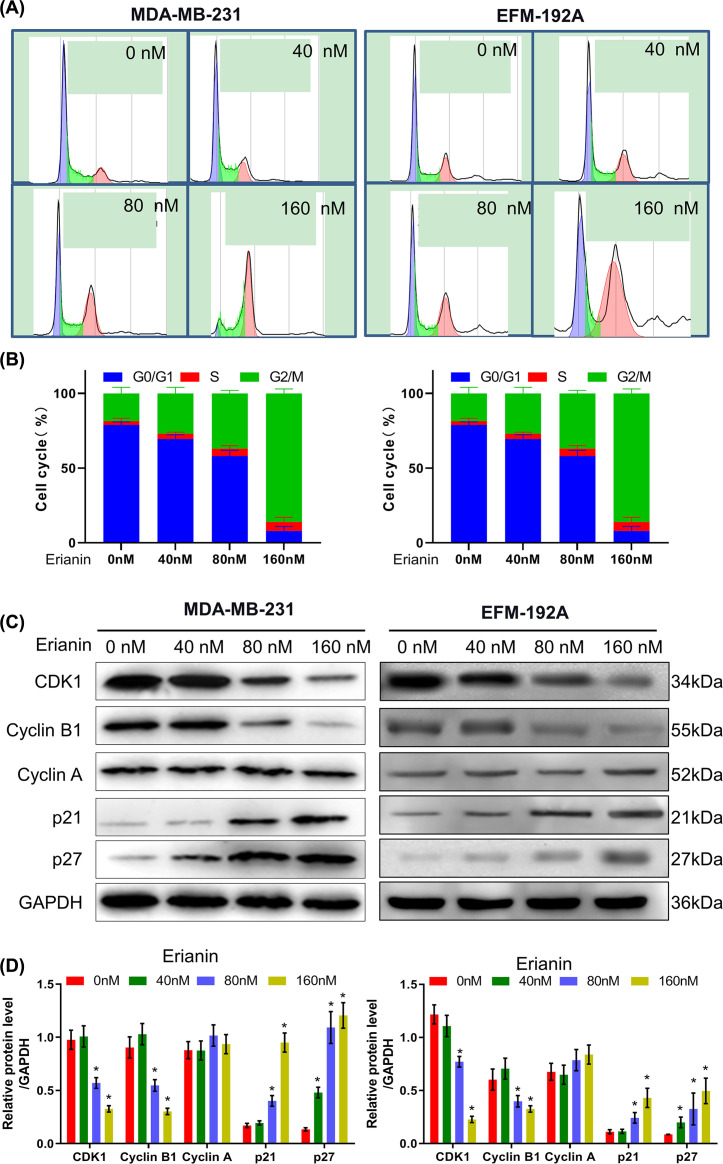
Erianin arrests cell cycle in G_2_/M phase and influences cell cycle-related protein expression in a dose-dependent way (**A**) Flow cytometry results of MDA-MB-231 and EFM-192A cells cycle distribution treated with erianin at different concentrations for 24 h. (**B**) Flow cytometry results of MDA-MB-231 and EFM-192A cells cycle quantification treated with erianin at different concentrations for 24 h. (**C**) Protein levels of cell cycle-related protein in MDA-MB-231 and EFM-192A cells treated with erianin at different concentrations for 24 h. (**D**) The histogram was obtained after quantification of the Western blotting results from (C). When compared with 0 nM group, * indicated *P*<0.05.

With the goal of exploring whether erianin could regulate cell cycle, we analyzed cell cycle distribution after 24 h erianin treatment at 40, 80, 160 nM by flow cytometry. Cell cycle results showed that erianin could significantly induce cell arrest in G_2_/M phase in a dose-dependent way (Figure 2A,B). Furthermore, as shown in Figure 2C,D, the expressions of CKI (p21, p27) were up-regulated significantly (*P*<0.05), while the expressions of CDK1 and Cyclin B1 were decreased significantly (*P*<0.05), with no significant changes in Cyclin A expression. These results confirmed that erianin could induce the cell cycle arrest in MDA-MB-231 by down-regulating CDK1 and Cyclin B1.

### Erianin can promote cell apoptosis and effect on apoptosis-related protein expression in a dose-dependent way

**Figure 3:**
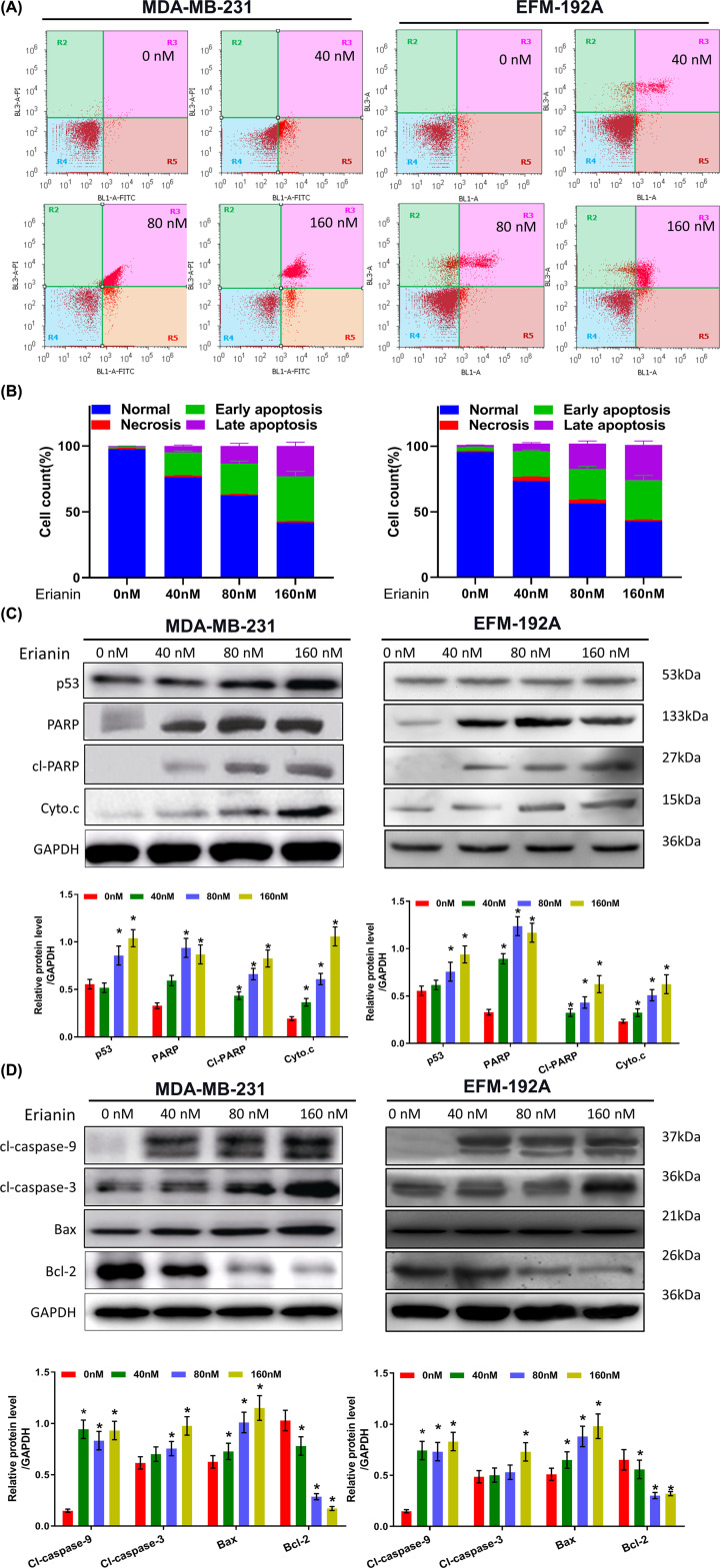
Erianin can promote cell apoptosis and effect on apoptosis-related protein expression in a dose-dependent way (**A**) Flow cytometry results of MDA-MB-231 and EFM-192A cells apoptosis distribution treated with erianin at different concentrations for 24 h. (**B**) Flow cytometry results of MDA-MB-231 and EFM-192A cells apoptosis quantification treated with erianin at different concentrations for 24 h. (**C**) Protein levels of apoptosis-related protein in MDA-MB-231 and EFM-192A cells treated with erianin at different concentrations for 24 h. (**D**) Histogram shows the quantitative results of (C)s. * indicated *P*<0.05.

To study the effect of erianin on apoptosis of breast cancer cells, we analyzed the apoptotic proportion with 40, 80, 160 nM erianin treatment for 24 h. MDA-MB-231 and EFM-192A cells apoptotic proportions at early and late stage were increased with erianin treatment in a dose-independent way, coincident with DAPI staining (Figure 3A,B). Besides, apoptosis-related protein expression, such as PARP, Cyto C, caspase 3/9, and Bcl-2 were measured. The expressions of Cyto C, PARP, p53, cl-Caspase3/9 (cleaved form of caspase-3 and caspase-9), Bax and other pro-apoptotic protein expression were up-regulated and anti-apoptotic protein Bcl-2 expression was down-regulated in MDA-MB-231 and EFM-192A cells (Figure 3C). The difference was statistically significant (Figure 3D). Cyto C is a protein that is specifically localized on the mitochondria, and its relative expression level increases significantly with the increased concentration of the erianin. The above data indicated that erianin could induce apoptosis in MDA-MB-231 and EFM-192A cells probably via mitochondrial pathway.

### Erianin activates PI3K/Akt pathway

**Figure 4:**
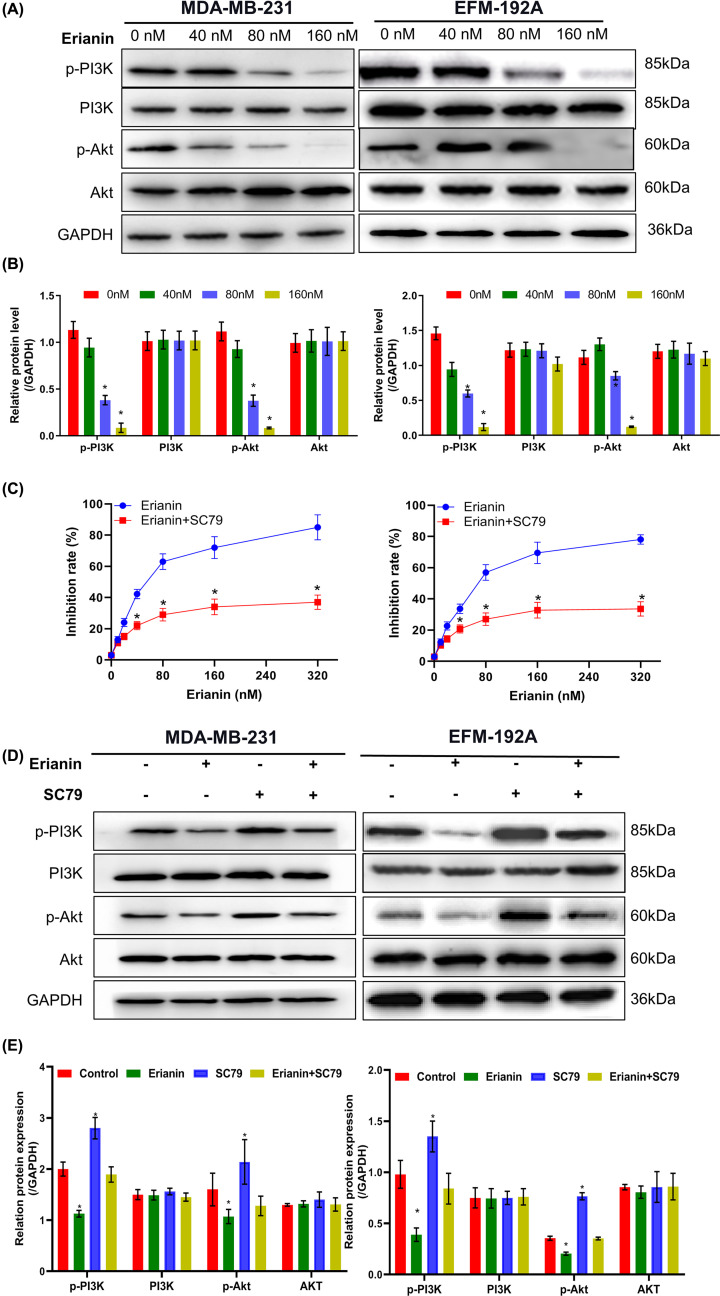
Erianin activates PI3K/Akt pathway (**A**) p-PI3K/p-Akt and total PI3K/Akt expression levels in MDA-MB-231 and EFM-192A cells treated with erianin at different concentrations for 24 h. (**B**) Histogram shows the quantitative results of (A). (**C**) MTT results of MDA-MB-231 and EFM-192A cells treated with erianin at different concentrations with or without Akt pathway agonists SC79. (**D**) The results of PI3K and AKT phosphorylation were detected by immunoblotting, and the bands were quantified (**E**). * indicated *P*<0.05. (**E**) The histogram was obtained after quantification of the Western blotting results from (D). When compared with control group, * indicated *P*<0.05.

p-PI3K, p-Akt expression was down-regulated with erianin treatment in a dose-dependent way, while the expression of total PI3K/Akt was unchanged (Figure 4A,B). Furthermore, incubated with Akt agonists SC79, the inhibition of cell proliferation with erianin treatment was decreased significantly in MTT assay, indicating activation of PI3K/Akt pathway was relevant to the antitumor effect of erianin (Figure 4C). Subsequently, the expression levels of p-AKT, AKT, p-PI3k, and PI3K in PI3K-related pathways in MDA-MB-231 and EFM-192A cells after adding SC79 were detected by immunoblotting, and it was found that the phosphorylation of AKT and PI3K could be up-regulated after SC79 alone levels, while in the group given both erianin and SC79, the inhibition of PI3K and Akt phosphorylation caused by erianin can be restored (Figure 4D,E), further confirming that the inhibition of PI3K/AKT pathway plays an important role in the cell inhibition induced by eranin. Taken together, these results indicate the activation of the PI3K/Akt pathway plays important role in erianin regulation on breast cancer.

## Discussion

Herbal medicine is one of the most traditional cultures in China, and derivation of the biologically active molecules from Chinese medicine plays important roles in drug screening. Erianin, derived from *Dendrobium*, has shown to have effects on different cellular processes, for example, activity in bacterial infections [24], and exert its anti-inflammatory effect in mouse model of ulcerative colitis [25] and diabetic nephropathy model [26]; moreover, it reduced high glucose-induced renal tubular epithelial cell damage [27]. Furthermore some studies have shown block blood vessel formation [21,28,29], while the among them, the anticancer activity of erianin was most reported [30], which could affect the proliferation, apoptosis, autophagy, metastasis and angiogenesis of cancer cells; the main pathways involved are the JNK, ERK, and p53. In the present study, we explored the effects and molecular mechanism of erianin in human TNBC cells.

First, MDA-MB-231 and EFM-192A cells proliferation was inhibited with erianin treatment in a dose-dependent way shown in MTT analysis. Secondly, typical apoptotic morphology (chromatin condensation and fracture) was detected in the MDA-MB-231 cells nucleus with erianin treatment for 24 h with DAPI staining, indicating erianin could induce MDA-MB-231 cells apoptosis. Thirdly, cell cycle results showed that erianin could significantly induce cell arrest in G_2_/M phase in a dose-dependent way. Cell cycle, a complex process of cell regulation, consists of cyclin-dependent kinase CDK, cyclin and cyclin-dependent kinase inhibitor (CDKI), regulating mutually and controlling cell cycle. Activation of CDK1 and Cyclin B1 is a necessity for the progression of G_2_/M. As putative CDKIs, p21 and p27 negatively regulate cell cycle progression, expressions of CKI (p21, p27) were up-regulated, and expressions of CDK1 and Cyclin B1 were down-regulated by Western analysis, coincident with flow cytometry analysis. These results suggest that erianin promoted cell cycle arrest in G_2_/M phase through up-regulating CDKI expression and down-regulating CDK expression.

Apoptosis ratio of MDA-MB-231 and EFM-192A cells at early and late stage were increased with erianin treatment in a dose-independent way, measured by flow cytometry. There are two pathways involved in cell apoptosis, endogenous and exogenous pathway. In endogenous pathway, the changes of mitochondrial membrane permeability are very significant under stimulation, followed by releasing cytochrome C into the cytoplasm. Combined cytochrome C with apoptosis protein activating factor 1 (Apaf-1) in the cytoplasm recruits Caspase-9 for Caspase cascade reaction. Subsequent activation of Caspase-3 can induce apoptosis by PARP splicing, which leads to the loss of the DNA repair capacity. On the other hand, the activation of p53 can also promote cell apoptosis. Up-regulated p53 can increase the expression of CDKI p21 and p27, blocking the cell cycle. Furthermore, up-regulated p53 induces the expression of apoptotic protein Bax and decreases the expression of anti-apoptotic protein Bcl-2, followed by activation of Caspase cascade reaction. The expression of these apoptosis-related proteins was measured in our study, which indicate that erianin can up-regulate the expression of p53 and induce MDA-MB-231 and EFM-192A cells apoptosis probably via mitochondrial pathway.

p-PI3K, p-Akt expression was down-regulated with erianin treatment in a dose-dependent way, while the expression of total PI3K/Akt was unchanged, indicating that erianin induced MDA-MB-231 and EFM-192A cells apoptosis through PI3K/Akt signaling pathway. Furthermore, after incubation with specific Akt agonists, the inhibitory effect of erianin treatment was abolished significantly. The cytotoxicity of erianin was decreased significantly when PI3K/Akt pathway was inhibited, indicating activation of PI3K/Akt pathway played an important role on the antitumor effect of erianin.

## Conclusion

In summary, the present study further confirmed that erianin, derived from traditional Chinese medicine *Dendrobium* significantly inhibited cell proliferation, promoted cell cycle arrest and apoptosis in human TNBC cells. Our findings demonstrate that erianin can arrest cell cycle in G_2_/M phase and activate mitochondrial pathway and inhibition of PI3K/Akt pathway to inhibit tumor growth. The inhibition of PI3K/Akt pathway by erianin treatment plays an important role in this process. Our study focused on the pharmacological effects and the molecular mechanism of erianin, providing a new potential chemotherapy drug for human breast cancer. Given the experimental conditions, the chemical structure of erianin, the direct targets, and precise molecular mechanism of erianin in human breast cancer need to be further identified.

## Data Availability

All data are included in the manuscripts，for the original data of the present study are available from the corresponding author on reasonable request.

## Abbreviations

CDKI: cyclin-dependent kinase inhibitor
DAPI: 4′,6-diamidino-2-phenylindole
HRP: Horseradish Peroxidase
MTT: 3-(4,5-Dimethylthiahiazo-2-yl)-2,5-diphenyltetrazolium bromide
PARP: Poly(ADP-Ribose) Polymerase
PI: Propidium iodide
TBST: Tris buffered saline +Tween20
TNBC: triple-negative breast cancer
