# Expression of Concern: Erianin induces triple-negative breast cancer cells apoptosis by activating PI3K/Akt pathway

**DOI:** 10.1042/BSR-2021-0093_EOC

**Published:** 2024-07-12

**Keywords:** breast cancer, cell apoptosis, cell proliferation, erianin, PI3K/Akt pathway

The Editorial Office has been made aware of potential issues surrounding the scientific validity of this paper, hence has issued an expression of concern to notify readers whilst the Editorial Office investigates. It has been noted that Figure 2C MDA-MB-231 GAPDH panel appears as Figure 4A EFM-192A PI3K panel, and Figure 2C MDA-MB-231 Cyclin B1 panel appears as Figure 4A MDA-MB-231 p-PI3K panel, both with vertical flipping and stretching transformation. Additionally, the Editorial Office has concerns surrounding similarities in Figure 2C MDA-MB-231 p21 panel and Figure 4A MDA-MB-231 p-Akt panel.

The authors were contacted regarding these concerns, and have offered explanations for the duplications between Figure 2C GAPDH and Figure 4A PI3K panels, as well as Figure 2C Cyclin B1 and Figure 4A p-PI3K panels. The authors state that due to mislabelling of files, similar images were accidentally used during figure assembly. However, the authors state Figure 2C p21 panel and Figure 4A p-Akt panel are from two independent experiments. The authors have provided replacement data for Figure 2C MDA-MB-231 GAPDH, Figure 2C MDA-MB-231 Cyclin B1, Figure 2C MDA-MB-231 p21, Figure 4A EFM-192A PI3K, Figure 4A MDA-MB-231 p-PI3K, and Figure 4A MDA-MB-231 p-Akt panels. However given the extent of the similarities identified between the figures, the Editorial Office is issuing an expression of concern.

